# Personality traits and adolescent anxiety: serial mediating roles of Red music cultural identification and self-identity

**DOI:** 10.3389/fpsyg.2026.1904643

**Published:** 2026-09-03

**Authors:** Qilin Huang, Shuxiang Hu, Ying Liu

**Affiliations:** 1 Academy of Music, Southwest University, Chongqing, China; 2 School of Psychology, Southwest University, Chongqing, China; 3 Mental Health Research Institute of Chinese Music, Southwest University, Chongqing, China

**Keywords:** adolescent anxiety, adolescent self-identity, big five personality, Red music cultural identification, serial mediation

## Abstract

**Objective:**

Adolescence is a critical period for personality formation and vulnerability to anxiety. Although personality traits are well-established predictors of adolescent anxiety, comparatively little research has examined the cultural mechanisms underlying this association. Drawing on culture–personality interaction theory, this study examined whether Red music cultural identification and self-identity sequentially mediate the association between Big Five personality traits and anxiety among Chinese adolescents. Specifically, we tested the pathway “Big Five personality traits → Red music cultural identification → self-identity → adolescent anxiety.”

**Methods:**

A cross-sectional questionnaire survey was conducted using data collected online from 1,751 Chinese primary and secondary school students (45.57% boys; Grades 4–12). Measures included the Chinese Big Five Personality Inventory–Brief Version, the Self-Rating Anxiety Scale, a self-developed Red Music Cultural Identification Scale, and the Adolescent Self-Identity Questionnaire. Serial mediation effects were examined using PROCESS Macro Model 6 with bias-corrected bootstrap confidence intervals based on 5,000 resamples.

**Results:**

Neuroticism significantly negatively predicted Red music cultural identification, whereas conscientiousness significantly positively predicted it. Red music cultural identification, in turn, significantly positively predicted adolescent self-identity, and self-identity significantly negatively predicted anxiety. Bootstrap analyses confirmed that the serial mediation pathway “personality traits → Red music cultural identification → self-identity → adolescent anxiety” was statistically significant.

**Discussion:**

These findings support the view that the association between personality traits and adolescent anxiety operates partly through culturally embedded identity processes. By identifying Red music cultural identification as a culturally specific psychological construct linked to self-identity, this study extends theoretical understanding of culture–personality interaction in adolescent development and offers a theoretical basis for exploring culturally grounded approaches to adolescent mental health intervention.

## 1 Introduction

Adolescence is a pivotal stage of physical and psychological development during which young people face the intertwined challenges of academic pressure, social adaptation, and self-identity formation (Erikson, 1968; Branje, 2022; Crocetti et al., 2023; Orben et al., 2024). Amid rapid economic and social transformation, monolithic standards of success and increasingly narrow systems of social evaluation may intensify developmental pressures and contribute to anxiety-related problems (Polanczyk et al., 2015; Dong et al., 2025). Adolescent anxiety has become a major global public mental health concern, with the global pooled prevalence of mental disorders reaching 13.4% and anxiety disorders accounting for 6.5% of adolescents worldwide (Polanczyk et al., 2015; GBD 2019 Mental Disorders Collaborators, 2022; Solmi et al., 2022). Similar trends have been observed in China, where the age-standardized prevalence of mental disorders among adolescents reached 8.9% in 2021 and nationwide surveys have reported anxiety detection rates of 20–30% (Liu et al., 2020; Dong et al., 2025). Among the many factors shaping adolescent anxiety, self-identity formation and cultural identification have emerged as particularly important psychological determinants: adolescents with unstable or poorly integrated self-concepts consistently report higher internalizing symptom levels, and the degree of cultural belonging plays a meaningful role in moderating this vulnerability (Crocetti et al., 2023). Amid multicultural influences and competing value systems, the cultural identification and self-identity development of Chinese adolescents face unprecedented challenges (Zeng et al., 2013; Ward and Szabó, 2023; Peng, 2025; Zhao, 2026), and identity instability shows a robust bidirectional association with internalizing problems (Crocetti et al., 2009; Luyckx et al., 2013; Maehler and Hernández-Torrano, 2025).

Personality traits are stable psychological factors consistently associated with mental health outcomes and are important intervention targets in adolescence (Anglim et al., 2020; Bleidorn et al., 2022; Roberts and Yoon, 2022; Ai et al., 2025). The Big Five framework (Costa and McCrae, 1992; Soto and John, 2017) conceptualizes stable individual differences across five dimensions: neuroticism (emotional instability and susceptibility to negative affect), extraversion (social engagement and positive affectivity), openness (imagination and esthetic sensitivity), agreeableness (trust and cooperation), and conscientiousness (self-regulation and achievement motivation) (Bleidorn et al., 2022). Among these, neuroticism and conscientiousness have consistently emerged as the strongest, oppositely directed predictors of anxiety (Kotov et al., 2010; Naragon-Gainey and Watson, 2018; Roberts and Yoon, 2022): neuroticism reflects heightened emotional reactivity and weaker regulatory capacity and is positively associated with anxiety (Goodwin et al., 2003; Gu et al., 2014), whereas conscientiousness reflects stronger self-regulation and confers protective effects on mental health (Friedman et al., 1993; Bienvenu et al., 2004; Ai et al., 2025). Although the direct effects of personality traits on anxiety are well established (Kotov et al., 2010; Roberts and Yoon, 2022), the mechanisms through which personality influences anxiety—particularly the role of the cultural environment—remain insufficiently understood (Lu et al., 2023; Kitayama and Salvador, 2024). Culture–personality interaction theory (Kardiner and Linton, 1945) posits that personality shapes emotional adaptation through ongoing interaction with cultural institutions; within this framework, the effects of personality on anxiety may be transmitted through individuals’ engagement with cultural institutions and their internalization of mainstream cultural values (Lu et al., 2023; Kitayama and Salvador, 2024).

Within the Chinese sociocultural context, the transmission of mainstream values is usually achieved through specific cultural symbols, for instance, Red music. Red music—referred to, in earlier Chinese musicology as revolutionary songs or revolutionary music—now has been examined by generations of Chinese musicologists and cultural historians as a genre encompassing works produced during the New Democratic Revolution, the socialist-construction period, the reform era, and the contemporary period, characterized by explicit ideological content and mass-participatory esthetic form (Hu and Hu, 2022; Han and Guo, 2023). In the 15th Five-Year Plan for National Economic and Social Development of the People’s Republic of China, red resources have also been emphasized to play a role in promoting the healthy development of young people (State Council of the People's Republic of China, 2026). Contemporary scholarship has consolidated Red music as a distinctive system of cultural symbols institutionally recognized for transmitting revolutionary history, patriotic sentiment, and collectivist values (Zhang, 2023; Zhang, 2024; He et al., 2025; Liu, 2025). Extending this conceptualization to the psychological level, Red music cultural identification is defined as an individual’s endorsement and internalization of Red music and the cultural values it conveys—a culturally specific form of musical identification rooted in China’s sociohistorical context (He et al., 2025). Music transforms abstract values and collective memories into perceptible affective experiences (DeNora, 2000; MacDonald et al., 2017), thus, the formation of Red music cultural identification is not a process of passive reception. Instead, individuals actively perceive cultural symbols, resonate with them emotionally, and integrate them cognitively on the basis of their stable personality traits (Hargreaves et al., 2002; Liu, 2025). Red music conveys collectivist and patriotic values whose internalization depends substantially on personality dispositions (McCrae and Costa, 1999; Lu et al., 2023), and when exposed to identical cultural stimuli, individuals with different personality profiles exhibit markedly different patterns of affective response and value internalization (Rentfrow and Gosling, 2003; Kitayama and Salvador, 2024). Accordingly, we propose:

*H1*: Different personality dimensions exert differential predictive effects on adolescents' Red music cultural identification.

Self-identity refers to the psychosocial process through which individuals explore and construct answers to the question “Who am I?” and constitutes a central developmental task in adolescence (Erikson, 1968; Branje, 2022). According to social identity theory (Tajfel and Turner, 1986; Ward and Szabó, 2023), individuals derive a sense of self from the social groups to which they belong, and group identification provides self-esteem, belonging, and security. Cultural identification, as a core form of social identity, is a major contributor to self-identity formation (Crocetti et al., 2023; Chen and Xu, 2026); within China’s collectivist context, it represents the deepest level of identification within the national community, with value-based identification at its core (Luo and Chen, 2026). From this perspective, Red music cultural identification is not merely an esthetic preference for a musical form but a process through which individuals internalize the historical memory, national spirit, and value orientations embedded in Red culture as part of the self (He et al., 2025). When individuals strongly identify with the values conveyed through Red music, this sense of cultural belonging may provide a stable value framework and sense of purpose, reinforcing their understanding of social roles and social responsibility and thereby promoting self-identity integration and consolidation (MacDonald et al., 2017; Luo and Chen, 2026). Hence, we propose:

*H2*: Red music cultural identification positively predicts adolescent self-identity.

Self-identity, in turn, functions as an important protective factor in adolescent mental health. Drawing on Erikson's (1968) identity theory and Deci and Ryan's (2000) self-determination theory—frameworks that continue to inform contemporary research on adolescent identity and well-being (Branje, 2022; Maehler and Hernández-Torrano, 2025)—individuals with coherent self-narratives and clearly defined value orientations tend to appraise external stressors more rationally, exhibiting lower anxiety (Wang and Yao, 2021). Conversely, identity diffusion reflects the absence of a stable self-referential framework and is associated with heightened threat sensitivity and intensified anxiety (Marcia, 1966; Zeng et al., 2013; Crocetti et al., 2023). Empirical work further confirms that self-identity clarity and coherence are important predictors of mental health, particularly in collectivist cultural contexts (Vignoles et al., 2006; Maehler and Hernández-Torrano, 2025). Thus, we propose:

*H3*: Self-identity negatively predicts adolescent anxiety.

Extensive research has examined each of the constructs relevant to the present study. Personality traits and adolescent anxiety have been linked across a wide range of samples, including Chinese adolescents (Gu et al., 2014; Anglim et al., 2020; Ai et al., 2025). Self-identity has been established as a robust predictor of adolescent internalizing problems (Branje, 2022; Maehler and Hernández-Torrano, 2025), and cultural identification has been increasingly recognized as a contributor to identity development in collectivist contexts (Ward and Szabó, 2023; Chen and Xu, 2026). Chinese adolescents’ engagement with musical culture—including popular, indie, and Western musical forms—has likewise been widely studied (Ye et al., 2025). While these separate lines of evidence are well established, three integrative questions warrant further investigation. First, prior work has examined the personality–identity link (Crocetti et al., 2023), the culture–personality link (Lu et al., 2023; Kitayama and Salvador, 2024), and the identity–anxiety link (Branje, 2022; Maehler and Hernández-Torrano, 2025) as separate dyads, but the sequential pathway through which personality effects on anxiety may be transmitted via culturally embedded identity processes has been examined in only a small number of studies and has not been systematically modeled within a single framework. Second, existing music–identity research has been dominated by studies of popular, Western, or genre-general musical engagement (MacDonald et al., 2017; de Witte et al., 2022; Ye et al., 2025), and although He et al. (2025) recently examined Red music identity in relation to subjective well-being, to our knowledge no prior empirical study has examined how identification with a culture-specific mainstream musical repertoire such as Red music—distinct from popular or Western music—shapes adolescent self-identity. Third, to our knowledge, personality traits, Red music cultural identification, self-identity, and adolescent anxiety have not been integrated within a single serial-mediation framework in the Chinese sociocultural context. Accordingly, the present study adopted the Big Five traits as core predictors and tested the serial mediation pathway “personality traits → Red music cultural identification → self-identity → adolescent anxiety” in a sample of Chinese primary and secondary school students. Rather than claiming that each component is unstudied, the study’s contribution lies in specifying how these constructs interlock through a culturally specific pathway. Accordingly, we propose:

*H4*: Red music cultural identification and self-identity jointly serve as significant serial mediators in the relationship between personality traits and anxiety.

## 2 Materials and methods

### 2.1 Participants

This study employed a cross-sectional survey design. Questionnaires were distributed through the *Wenjuanxing* online survey platform to primary and secondary schools across multiple Chinese provinces, with survey links forwarded to students and parents by school staff. Participation was voluntary, and no incentives were offered. The study was approved by the Ethics Committee of the Department of Psychology, Southwest University. Participants were informed of the data collection procedures and confidentiality measures and were assured that all information would be used solely for research purposes.

A total of 2,000 questionnaires were collected. After 249 invalid responses containing missing values were excluded, 1,751 valid questionnaires were retained, yielding a valid response rate of 87.55%. The final sample comprised 798 boys (45.57%) and 953 girls (54.43%), covering the developmental period from late childhood to late adolescence (approximately 9–18 years of age based on the Chinese school system). Specifically, the sample included 614 students in Grades 4–6 of elementary school (35.10%; approximate age range 9–12 years), 731 students in Grades 7–9 of junior high school (41.75%; approximate age range 12–15 years), and 406 students in Grades 10–12 of senior high school (23.19%; approximate age range 15–18 years).

### 2.2 Measures

#### 2.2.1 Big five personality inventory

The study employed the Chinese Big Five Personality Inventory–Brief Version developed by Wang et al. (2011), which has continued to be applied in recent Chinese adolescent and young adult samples (Ai et al., 2025). The scale comprises 40 items across five dimensions—neuroticism, conscientiousness, agreeableness, openness, and extraversion—with each dimension measured by 8 items. Items are rated on a 6-point Likert scale ranging from 1 (strongly disagree) to 6 (strongly agree). The brief version has demonstrated sound psychometric properties. Internal consistency coefficients (Cronbach’s α) ranged from 0.764 (agreeableness) to 0.814 (neuroticism), with a mean of 0.793. Test–retest reliability coefficients across a 10-week interval ranged from 0.672 (agreeableness) to 0.811 (openness), with a mean of 0.742. Exploratory factor analysis supported a clear five-factor structure, with the five factors accounting for 43.49% of the total variance, and confirmatory factor analysis further confirmed the adequacy of the model fit. In addition, correlations between the brief version and corresponding dimensions of the full-length CBF-PI exceeded 0.85, and correlations with corresponding dimensions of widely used Big Five measures, including the NEO-PI-R and BFI, were also significant. Overall, the scale provides a concise and effective assessment of the Big Five personality traits.

#### 2.2.2 Red Music Cultural Identification Scale

Building on an extensive literature review, preliminary qualitative work, and established theoretical frameworks, we developed the Red Music Cultural Identification Scale. The scale was constructed by integrating the specific connotations of Red music culture with reference to the Brief General Cultural Identification Questionnaire developed by Huang and Bi (2021), the Cultural Confidence Questionnaire developed by Zhou and Bi (2020), and the core dimensions and item formulations of He et al.’s (2025) Red Music Identity Scale — a recently developed instrument that itself illustrates the continued application of these questionnaire-development approaches to Chinese cultural-identification research. Recent neurophysiological evidence further supports the psychological reality of Red music engagement: He et al. (2026), using the Single-Category Implicit Association Test combined with event-related potentials, demonstrated that Red song exposure elicits distinct neural correlates across multiple processing stages (N1, P2, N3, and LPC components), indicating that Red music engagement is not merely a cultural or ideological construct but has measurable psychophysiological correlates in the Chinese context. The resulting scale comprises four dimensions—cognitive identification (“I can clearly explain to others what Red music is”), affective identification (“The emotions expressed in Red music resonate with my own emotional experiences”), volitional identification (“When I face difficulties or challenges, the indomitable will embodied in Red music inspires me”), and behavioral-intention identification (“When my school holds activities related to Red music, I am willing to participate”)—with a total of 30 items. The scale provides a systematic assessment of individuals’ identification with Red music culture. Respondents answered on a 7-point Likert scale ranging from 1 (strongly disagree) to 7 (strongly agree).

During the scale development, five experts were invited to conduct a content validity review. Three of these experts came from the fields of music education and red culture research, and two from psychology and psychometrics. All possessed substantial publication records and extensive expertise in identity research in the Chinese cultural context. In addition, to ensure conceptual clarity and cultural relevance, the research team conducted focus group discussions with the members of the target population.

To avoid the inflation of model fit that arises when EFA and CFA are conducted on the same data, we randomly partitioned the total sample (*N* = 1,751) into two independent subsamples: Subsample 1 (n₁ = 875) for exploratory factor analysis (EFA) and Subsample 2 (n₂ = 876) for confirmatory factor analysis (CFA). The two subsamples did not differ significantly in gender, age, or grade level (all *p* > 0.05). The item screening described below served as a psychometric validation exercise only; all subsequent substantive analyses used total scores from the original 30-item scale.

Prior to factor extraction, the suitability of the data for exploratory factor analysis was assessed using two established criteria. The Kaiser–Meyer–Olkin (KMO) measure of sampling adequacy assesses whether the correlation matrix contains sufficient common variance for factor extraction, with values ≥ 0.80 considered meritorious (Kaiser, 1974). Bartlett’s test of sphericity evaluates whether the correlation matrix differs significantly from an identity matrix, confirming sufficient shared variance to justify factor analysis (Bartlett, 1954). Principal component analysis was then performed, and factor loadings were rotated using Varimax orthogonal rotation, which yields a simple, interpretable factor structure when the underlying factors are theoretically expected to be uncorrelated or minimally correlated (Kaiser, 1958). These procedures remain standard in contemporary scale-development practice (Hair et al., 2022; Kline, 2023).

Exploratory factor analysis: in Subsample 1, the Kaiser–Meyer–Olkin measure was 0.973 and Bartlett’s test of sphericity was significant, χ^2^(435) = 25,341.79, *p* < 0.001. Principal component analysis with Varimax rotation extracted four factors with eigenvalues greater than one, accounting for 72.85% of the total variance and corresponding to the hypothesized structure of cognitive, affective, volitional, and behavioral-intention identification.

Inspection of the rotated factor loading matrix (Table 1) identified eight items with cross-loadings ≥ 0.40 (A1, A7, A9, A10 in cognitive identification; B6–B9 in affective identification), which violated the simple-structure principle (Thurstone, 1947). Following the item-retention criteria of Hair et al. (2010) and Wu (2010), which remain widely applied in current psychometric research (Hair et al., 2022), these items were provisionally removed for validation purposes. The remaining 22 items exhibited a clean factor structure, with each item loading above 0.60 only on its intended factor.

**Table 1:** Rotated factor loading matrix of the Red Music Cultural Identification Scale (EFA, Subsample 1, n_1_ = 875).

Item	Factor 1	Factor 2	Factor 3	Factor 4
A1	0.580	0.535		
A2	0.708			
A3	0.791			
A4	0.795			
A5	0.683			
A6	0.763			
A7	0.682		0.413	
A8	0.751			
A9	0.660		0.498	
A10	0.655		0.400	
A11	0.711			
B1		0.660		
B2		0.707		
B3		0.750		
B4		0.773		
B5		0.719		
B6	0.538	0.541		
B7	0.474	0.620		
B8	0.526	0.589		
B9	0.540	0.584		
C1			0.708	
C2			0.716	
C3			0.773	
C4			0.699	
C5			0.773	
D1				0.679
D2	0.457			0.719
D3				0.742
D4				0.708
D5				0.708

Extraction method: principal component analysis (PCA). Rotation method: Varimax rotation with Kaiser normalization. Rotation converged after six iterations. Factor loadings ≥0.40 are displayed.

Confirmatory factor analysis: the 22-item four-factor model was tested in Subsample 2. All standardized factor loadings were significant and ranged from 0.659 to 0.892, exceeding the 0.60 threshold. The model demonstrated an acceptable fit to the data (Table 2), satisfying the criteria of Hu and Bentler (1999), which continue to serve as a benchmark in current SEM practice (Kline, 2023), and successfully cross-validated the four-dimensional structure in an independent subsample.

**Table 2:** Model fit indices for the four-factor CFA model of the Red Music Cultural Identification Scale (Subsample 2, n_2_ = 876).

χ2	df	χ^2^/df	GFI	AGFI	CFI	TLI	NFI	RMSEA	RMR
1204.142	203	5.932	0.882	0.854	0.932	0.923	0.920	0.075	0.112

χ^2^, chi-square statistic; df, degrees of freedom; χ^2^/df, normed chi-square; GFI, Goodness-of-Fit Index; AGFI, Adjusted Goodness-of-Fit Index; CFI, Comparative Fit Index; TLI, Tucker–Lewis Index; NFI, Normed Fit Index; RMSEA, Root Mean Square Error of Approximation; RMR, Root Mean Square Residual. The four-factor CFA model demonstrated an acceptable fit to the observed data (CFI = 0.932, TLI = 0.923, RMSEA = 0.075).

Reliability: in the full sample (*N* = 1,751), the overall internal consistency coefficient of the 30-item scale used in all subsequent substantive analyses was excellent (Cronbach’s *α* = 0.946). Cronbach’s α coefficients for the four subscales were 0.962 for cognitive identification (11 items), 0.933 for affective identification (9 items), 0.923 for volitional identification (5 items), and 0.811 for behavioral-intention identification (5 items), all substantially exceeding the 0.70 threshold recommended by Nunnally (1978) and Hair et al. (2022). Combined with the split-sample EFA and CFA evidence reported above, these results indicate that the Red Music Cultural Identification Scale possesses satisfactory psychometric properties for use in the present study.

#### 2.2.3 Adolescent self-identity questionnaire

This study assessed participants’ self-identity using a self-developed Adolescent Self-Identity Questionnaire. The questionnaire was constructed primarily based on the survey framework for adolescent national identity proposed by Zeng et al. (2013) and the theoretical model of scientific identity developed by Wang and Yao (2021). It was aligned with the recent developmental identity-research literature (He et al., 2025), while also considering the developmental characteristics and social roles of adolescents. The questionnaire consists of 30 items distributed across four dimensions—identity cognition (“As a student, I clearly know that my primary task is to study”), role conflict and stress (“I feel a great deal of pressure to perform well across all subjects simultaneously”; reverse-scored), emotional belonging and commitment (“I have a sense of belonging to my school”), and behavioral engagement and consistency (“In class I listen attentively, and I take the initiative to preview and review my lessons”)—designed to comprehensively evaluate the integration of adolescents’ student identity across cognitive, affective, and behavioral domains. All items are rated on a 7-point Likert scale, with 1 representing “does not describe me at all” and 7 representing “describes me completely.”

To ensure methodological consistency, the Adolescent Self-Identity Questionnaire was subjected to the same split-sample cross-validation framework. The item screening below served as a psychometric validation exercise only; all subsequent substantive analyses used total scores from the original 30-item questionnaire.

Exploratory factor analysis: the same procedures were applied as described in Section 2.2.2. In Subsample 1, the Kaiser–Meyer–Olkin measure was 0.959 and Bartlett’s test of sphericity was significant, χ^2^(435) = 17,239.87, *p* < 0.001. Principal component analysis with Varimax rotation extracted four factors with eigenvalues greater than one, accounting for 63.01% of the total variance, and mapping onto the hypothesized dimensions of identity cognition, emotional belonging and commitment, role conflict and stress, and behavioral engagement and consistency.

Examination of the rotated factor loading matrix (Table 3) revealed six items with cross-loadings ≥ 0.40. Following Hair et al. (2010) and Wu (2010), items A9–A11 (identity cognition) and D5, D7, D10 (behavioral engagement and consistency) were provisionally removed for validation purposes.

**Table 3:** Rotated factor loading matrix of the Adolescent Self-Identity Questionnaire (EFA, subsample 1, n_1_ = 875).

Item	Factor 1	Factor 2	Factor 3	Factor 4
A1	0.708			
A2	0.687			
A3	0.739			
A4	0.801			
A5	0.773			
A6	0.762			
A7	0.655			
A8	0.716			
A9	0.604	0.521		
A10	0.546	0.450		
A11	0.588	0.558		
B1			0.721	
B2			0.711	
B3			0.732	
B4			0.706	
B5			0.749	
C1				0.729
C2				0.768
C3				0.829
C4				0.724
D1		0.685		
D2		0.732		
D3		0.766		
D4		0.758		
D5	0.491	0.615		
D6		0.766		
D7	0.493	0.578		
D8		0.639		
D9		0.687		
D10	0.457	0.657		

Extraction method: principal component analysis (PCA). Rotation method: Varimax rotation with Kaiser normalization. Rotation converged after six iterations. Factor loadings ≥0.40 are displayed.

A content-level review of the identity cognition dimension further identified a means–ends hierarchical relationship between items A2 (“I have relatively clear goals for my future…”) and A3 (“In order to achieve my future goals, I know how to plan my daily study and life”). The stem of A3 explicitly references the goals measured in A2, indicating that the two items are not parallel indicators but hierarchically ordered sub-components of a Future Self-Planning facet within identity cognition. Following the representative indicator approach (Kline, 2015; Little et al., 2002; Kline, 2023), A2 was retained as the higher-order indicator in the CFA model, with the execution-level content of A3 preserved at the construct level. This yielded a 23-item four-factor structure for validation.

Confirmatory factor analysis: the 23-item four-factor model was tested in Subsample 2. All standardized factor loadings ranged from 0.584 to 0.870, exceeding the 0.50 threshold (Hair et al., 2010). The model demonstrated acceptable fit (Table 4), satisfying the criteria of Hu and Bentler (1999) and cross-validating the four-dimensional structure in an independent subsample.

**Table 4:** Model fit indices for the four-factor CFA model of the Adolescent Self-Identity Questionnaire (subsample 2, n_2_ = 876).

χ2	df	χ^2^/df	GFI	AGFI	CFI	TLI	NFI	RMSEA	RMR
1231.021	224	5.496	0.870	0.840	0.905	0.892	0.886	0.072	0.167

χ^2^, chi-square statistic; df, degrees of freedom; χ^2^/df, normed chi-square; GFI, Goodness-of-Fit Index; AGFI, Adjusted Goodness-of-Fit Index; CFI, Comparative Fit Index; TLI, Tucker–Lewis Index; NFI, Normed Fit Index; RMSEA, Root Mean Square Error of Approximation; RMR, Root Mean Square Residual. The four-factor CFA model demonstrated an acceptable fit to the observed data (CFI = 0.905, TLI = 0.892, RMSEA = 0.072).

Convergent and discriminant validity: CR values ranged from 0.799 to 0.905, all exceeding 0.70. AVE values ranged from 0.444 to 0.580, with three of four dimensions surpassing the 0.50 benchmark (Fornell and Larcker, 1981), a criterion that continues to be applied in contemporary discriminant-validity practice (Hair et al., 2022). The AVE of emotional belonging and commitment (0.444) fell slightly below this threshold, likely reflecting the multifaceted nature of this construct—which encompasses feelings of belonging, emotional commitment, and affective evaluation of identity—for which a single factor is often insufficient to capture all variance. Given that its CR (0.799) substantially exceeded the 0.60 minimum, this dimension was retained. In the full sample (*N* = 1,751), the overall internal consistency coefficient of the 30-item questionnaire used in all subsequent substantive analyses was Cronbach’s *α* = 0.904, indicating excellent internal consistency (Nunnally 1978; Hair et al., 2022). Together with the split-sample EFA/CFA evidence and the CR/AVE indicators reported above, these results support the psychometric adequacy of the Adolescent Self-Identity Questionnaire for use in the present study.

#### 2.2.4 Self-rating anxiety scale

This study assessed the participants’ anxiety symptom levels using the Self-Rating Anxiety Scale (SAS). Originally developed by Zung (1971), the SAS is a widely used self-report instrument for measuring anxiety, with psychometric properties supported in recent evaluations (Vu et al., 2023). This scale comprises 20 items that primarily assess an individual’s subjective anxiety experience over the preceding week. The item content covers two aspects of anxiety: affective experiences (for example, fear and panic) and somatic symptoms (for example, heart palpitations, dizziness, and sleep disturbances). Items were rated on a 4-point scale ranging from 1 (none or a little of the time) to 4 (most or all of the time). The raw score was obtained by summing the responses across all items, and the standard score was then calculated by multiplying the raw score by 1.25 and taking the integer portion. According to Chinese norms, a standard score ≥ 50 is generally considered indicative of anxiety symptoms: scores of 50–59 reflect mild anxiety, 60–69 moderate anxiety, and 70 or above severe anxiety. The scale demonstrates sound psychometric properties; applications of the Chinese version of the SAS in domestic populations have shown good internal consistency and reliability and the ability to effectively distinguish individuals with anxiety disorders from the general population. Accordingly, the SAS is well-suited for the rapid preliminary screening and assessment of individuals’ anxiety levels.

### 2.3 Statistical analysis

All analyses were conducted with SPSS 31.0, and confirmatory factor analyses of the two self-developed instruments were performed in Amos 26.0. Descriptive statistics, correlation analyses, and reliability analyses were performed prior to hypothesis testing. In the present analyses, neuroticism (X₁) and conscientiousness (X₂) served as independent variables, Red Music Cultural Identification (M₁) and adolescent self-identity (M₂) as sequential mediators, and adolescent anxiety (SAS) as the dependent variable, specifying the serial pathway X → M₁ → M₂ → Y. We conducted mediation analyses using the PROCESS macro (Model 6), following the procedures described by Fang et al. (2012) and Hayes (2022), to examine the sequential mediation pathways from personality traits to Red music cultural identification, adolescent self-identity, and adolescent anxiety. Consistent with the psychometric strategy described above, all substantive analyses were based on the total scores of the original 30-item Red Music Cultural Identification Scale and the original 30-item Adolescent Self-Identity Questionnaire. All variables were standardized prior to analysis, and the significance of regression coefficients was tested using the bootstrap method with 5,000 resamples.

## 3 Results

### 3.1 Common method bias test

Since all variables in the present study were assessed using self-report questionnaires administered at a single time point, common method bias is a potential concern. At the data collection stage, several procedural controls including anonymous responses, the use of reverse-scored items in selected scales, and randomized item ordering were implemented to mitigate this risk. Harman’s single-factor test (Podsakoff et al., 2003), a technique whose limitations and appropriate interpretation continue to be discussed in contemporary methodological reviews (Podsakoff et al., 2024), was conducted by submitting all items to an unrotated exploratory factor analysis. The results yielded 19 factors with eigenvalues greater than 1, accounting for a cumulative 64.59% of the variance, with the first factor explaining 24.10% of the variance, which is well below the 40% threshold. These findings indicate that common method bias did not pose a serious concern in the present study, supporting the validity of the subsequent analyses.

### 3.2 Descriptive statistics and correlation analysis

The basic characteristics of the samples are listed in Table 5. A total of 1,751 adolescents were included in the study (798 boys, 45.57%; 953 girls, 54.43%). With respect to educational level, 614 participants (35.10%) were in grades 4–6 of elementary school, 731 (41.75%) were in grades 7–9 of junior high school, and 406 (23.19%) were in grades 10–12 of senior high school.

**Table 5:** Basic demographic characteristics of the sample *N* = 1,751.

Variable	Frequency	Percentage	Age (years)
Male	798	45.57%	9–18
Female	953	54.43%	9–18
Grades 4–6 (Elementary School)	614	35.10%	9–12
Grades 7–9 (Junior High School)	731	41.75%	12–15
Grades 10–12 (Senior High School)	406	23.19%	15–18

Approximate age ranges are derived from the standard Chinese school system.

Descriptive statistics for the main study variables are presented in Table 6. With respect to the Big Five personality dimensions, the means and medians for neuroticism (*M* = 22.65, SE = 0.22, SD = 9.11), conscientiousness (*M* = 32.80, SE = 0.20, SD = 8.39), agreeableness (*M* = 34.11, SE = 0.17, SD = 6.97), openness (*M* = 33.80, SE = 0.20, SD = 8.29), and extraversion (*M* = 32.09, SE = 0.18, SD = 7.46) were closely aligned, and the score distributions were broadly symmetric with no marked skewness. Agreeableness exhibited the smallest standard deviation (SD = 6.97), indicating the most concentrated scores across the sample, whereas neuroticism showed the largest standard deviation (SD = 9.11), reflecting greater individual differences in this dimension. With respect to adolescent self-identity, scores averaged *M* = 151.03 (SE = 0.70, SD = 29.12), and Red music cultural identification scores averaged *M* = 147.87 (SE = 0.71, SD = 29.63), with medians of 151 and 150, respectively. With respect to mental health, participants’ scores on the self-rated anxiety scale (SAS) averaged *M* = 44.87 (SE = 0.26, SD = 10.77), with a median of 44.

**Table 6:** Descriptive statistics for the main study variables *N* = 1,751.

Variable	*M*	SE	Mdn	SD
Neuroticism	22.65	0.22	22	9.11
Conscientiousness	32.80	0.20	32	8.39
Agreeableness	34.11	0.17	34	6.97
Openness	33.80	0.20	34	8.29
Extraversion	32.09	0.18	32	7.46
Self-identity	151.03	0.70	151	29.12
Red Music Cultural Identification	147.87	0.71	150	29.63
Anxiety (SAS)	44.87	0.26	44	10.77

Analyses of data from 1,751 adolescents (Table 7) revealed systematic associations between the Big Five personality traits, cultural identification, and anxiety. Neuroticism was significantly negatively correlated with conscientiousness (*r* = −0.345, *p* < 0.001), agreeableness (*r* = −0.207, *p* < 0.001), extraversion (*r* = −0.208, *p* < 0.001), and adolescent self-identity (*r* = −0.545, *p* < 0.001), but was strongly positively correlated with anxiety (*r* = 0.680, *p* < 0.001). The four adaptive personality traits—conscientiousness, agreeableness, openness, and extraversion—were not only moderately positively intercorrelated but also showed significant positive correlations with both adolescent self-identity and Red music cultural identification; self-identity and Red music cultural identification were themselves strongly positively correlated (*r* = 0.604, *p* < 0.001).

**Table 7:** Pearson correlations among study variables *N* = 1,751.

Variable	Neuroticism	Conscientiousness	Agreeableness	Openness	Extraversion	Self-identity	Red Music Cultural Identification
Conscientiousness	−0.345***	—					
Agreeableness	−0.207***	0.423***	—				
Openness	−0.077**	0.449***	0.375***	—			
Extraversion	−0.208***	0.377***	0.359***	0.515***	—		
Self-Identity	−0.545***	0.618***	0.458***	0.389***	0.411***	—	
Red Music Cultural Identification	−0.216***	0.471***	0.422***	0.433***	0.380***	0.604***	—
Anxiety (SAS)	0.680***	−0.347***	−0.262***	−0.128***	−0.230***	−0.540***	−0.250***

*p < 0.05, **p < 0.01, ***p < 0.001.

Both adaptive personality traits and the two forms of identification were significantly and negatively associated with anxiety. Overall, the correlation matrix points to a potential theoretical pathway: adaptive personality traits (for example, high conscientiousness and agreeableness) may foster cultural identification and self-identity, thereby providing psychological resources that help alleviate anxiety; conversely, high levels of neuroticism may weaken adolescents’ cultural and self-identity, contributing to the intensification of anxiety symptoms.

To address demographic transparency, Table 8 presents means and standard deviations of the main study variables broken down by sex and by school stage. Patterns across sex were highly comparable, with mean differences below one point on all five variables. Across school stages, developmentally plausible age-related trends emerged — a gradual rise in neuroticism and anxiety, and a modest decline in conscientiousness and self-identity from Grades 4–6 through Grades 10–12 — patterns consistent with the well-documented developmental literature on adolescent internalizing symptoms and identity exploration. Notably, mean scores on Red Music Cultural Identification remained stable across school stages (range: 146.51–148.69), supporting the age-invariance of this cultural construct within the studied developmental window.

**Table 8:** Means and standard deviations of main study variables by sex and school stage (*N* = 1,751).

Variable	Male (*n* = 798) M (SD)	Female (*n* = 953) M (SD)	Grades 4–6 (*n* = 614) M (SD)	Grades 7–9 (*n* = 731) M (SD)	Grades 10–12 (*n* = 406) M (SD)
Neuroticism	22.37 (9.17)	22.88 (9.07)	21.01 (9.02)	22.60 (9.15)	25.21 (8.62)
Conscientiousness	32.87 (8.15)	32.73 (8.59)	34.32 (8.73)	32.69 (8.23)	30.69 (7.66)
Red Music Cultural Identification	147.07 (30.17)	148.54 (29.18)	147.79 (32.57)	148.69 (27.97)	146.51 (27.87)
Self-identity	151.74 (29.24)	150.44 (29.03)	155.89 (30.10)	150.49 (29.43)	144.68 (25.62)
Anxiety (SAS)	44.63 (10.97)	44.83 (10.61)	43.52 (10.72)	44.52 (10.95)	46.99 (10.20)

SAS, Self-Rating Anxiety Scale. Values in parentheses are standard deviations. Approximate age ranges for school stages: Grades 4–6 (9–12 years), Grades 7–9 (12–15 years), Grades 10–12 (15–18 years), based on the standard Chinese school system.

### 3.3 Test of the serial mediation models

Neuroticism and conscientiousness have been consistently identified in previous meta-analyses as the two most robust personality predictors of anxiety (Kotov et al., 2010; Naragon-Gainey and Watson, 2018), a conclusion reinforced by recent global evidence linking anxiety onset to early adulthood (Solmi et al., 2022), and the two traits operate in opposing directions within emotional regulation. Therefore, the present study constructed two separate serial mediation models using neuroticism and conscientiousness as antecedent variables, with Red music cultural identification (M1) and adolescent self-identity (M2) as sequential mediators, and anxiety (Y) as the outcome variable. The models were estimated using PROCESS Macro Model 6, with bias-corrected 95% confidence intervals computed from 5,000 bootstrap resamples to examine the differences between the two personality traits in the underlying mediating mechanisms.

The regression analyses (Table 9) revealed that neuroticism significantly negatively predicted both Red music cultural identification (*β* = −0.216, *p* < 0.001) and adolescent self-identity (*β* = −0.435, *p* < 0.001). Red music cultural identification, in turn, significantly positively predicted self-identity (*β* = 0.510, *p* < 0.001). When both mediators were included in the model, the direct positive effect of neuroticism on anxiety remained significant (*β* = 0.545, *p* < 0.001), whereas self-identity exerted a significant negative effect on anxiety (*β* = −0.256, *p* < 0.001).

**Table 9:** Regression analysis for the serial mediation model with Neuroticism as the predictor.

Predictor	Outcome variable	*SE*	*β*	*t*	*p*	*R^2^*	*F*
Neuroticism	Red Music Cultural Identification	0.076	−0.216	−9.26	< 0.001	0.047	85.76
	Self-identity	0.053	−0.435	−26.29	< 0.001	0.545	1045.13
	Anxiety	0.024	0.545	26.74	< 0.001	0.503	590.41
Red Music Cultural Identification	Anxiety	0.008	0.023	1.05	0.293	0.503	590.41
	Self-identity	0.016	0.510	30.84	< 0.001	0.545	1045.13
Self-identity	Anxiety	0.009	−0.256	−10.25	< 0.001	0.503	590.41

R^2^ and *F* values pertain to the full regression equation in which the coefficient is estimated. Coefficients sharing the same R^2^ and F are drawn from the same equation with all predictors entered simultaneously. Specifically, three regression equations were estimated: (a) Red Music Cultural Identification regressed on Neuroticism (R^2^ = 0.047); (b) Self-Identity regressed on Neuroticism and Red Music Cultural Identification (R^2^ = 0.545); and (c) Anxiety regressed on Neuroticism, Red Music Cultural Identification, and Self-Identity (R^2^ = 0.503).

The bootstrap mediation analysis (Table 10) showed that the total effect of neuroticism on anxiety was significant and positive (*β* = 0.680, *p* < 0.001). The direct effect (*β* = 0.545) accounted for 80.1% of the total effect, whereas the total indirect effect was significant (*β* = 0.135, 95% CI [0.108, 0.160]) and accounted for the remaining 19.9%, indicating that Red music cultural identification and self-identity jointly functioned as partial mediators. Among the three indirect pathways, Path 1 (neuroticism → Red music cultural identification → anxiety) was not significant (*β* = −0.005, 95% CI [−0.014, 0.004]), indicating that Red music cultural identification did not exert a direct mediating effect on anxiety independent of self-identity. By contrast, Path 2 (neuroticism → self-identity → anxiety) was significant (*β* = 0.111, 95% CI [0.087, 0.134]) and showed the largest indirect effect. Notably, the serial mediation pathway, Path 3 (neuroticism → Red music cultural identification → self-identity → anxiety), was likewise significant (*β* = 0.028, 95% CI [0.020, 0.038]), demonstrating that Red music cultural identification does contribute to the indirect effect—but through its downstream influence on self-identity rather than through a direct route to anxiety.

**Table 10:** Bootstrap test of the mediation effects in the neuroticism model.

Pathway		*β*	SE	95% CI Lower	95% CI Upper	Significance
Path 1	X1 → M1 → Y (Ind1)	−0.005	0.005	−0.014	0.004	n.s.
Path 2	X1 → M2 → Y (Ind2)	0.111	0.012	0.087	0.134	*
Path 3	X1 → M1 → M2 → Y (Ind3)	0.028	0.005	0.020	0.038	*
	Total indirect effect	0.135	0.013	0.108	0.160	*
	Direct effect	0.545	—	—	—	*
	Total effect	0.680	—	—	—	*

X1, Neuroticism; M1, Red Music Cultural Identification; M2, Self-Identity; Y, Anxiety; *β*, standardized effect estimate; n.s., not significant. Confidence intervals were obtained from 5,000 bias-corrected bootstrap resamples. * indicates a significant effect (95% CI does not include zero).

The regression analyses (Table 11) showed that conscientiousness significantly positively predicted both Red music cultural identification (*β* = 0.471, *p* < 0.001) and adolescent self-identity (*β* = 0.429, *p* < 0.001), and that Red music cultural identification significantly positively predicted self-identity (*β* = 0.402, *p* < 0.001). In the full model including both mediators, the direct effect of conscientiousness on anxiety was not significant (*β* = −0.042, *p* = 0.103). Self-identity significantly negatively predicted anxiety (*β* = −0.590, *p* < 0.001), whereas Red music cultural identification showed a small positive residual effect on anxiety after controlling for conscientiousness and self-identity (*β* = 0.126, *p* < 0.001).

**Table 11:** Regression analysis for the serial mediation model with conscientiousness as the predictor.

Predictor	Outcome variable	SE	*β*	*t*	*p*	*R^2^*	*F*
Conscientiousness	Red Music Cultural Identification	0.074	0.471	22.36	< 0.001	0.222	499.84
	Self-Identity	0.066	0.429	22.52	< 0.001	0.507	900.40
	Anxiety	0.033	−0.042	−1.63	0.103	0.301	251.04
Red Music Cultural Identification	Anxiety	0.01	0.126	4.95	< 0.001	0.301	251.04
	Self-Identity	0.019	0.402	21.1	< 0.001	0.507	900.40
Self-Identity	Anxiety	0.011	−0.590	−20.69	< 0.001	0.301	251.04

R^2^ and F values pertain to the full regression equation in which the coefficient is estimated. Coefficients sharing the same R^2^ and F are drawn from the same equation with all predictors entered simultaneously. Specifically, three regression equations were estimated: (a) Red Music Cultural Identification regressed on Conscientiousness (R^2^ = 0.222); (b) Self-Identity regressed on Conscientiousness and Red Music Cultural Identification (R^2^ = 0.507); and (c) Anxiety regressed on Conscientiousness, Red Music Cultural Identification, and Self-Identity (R^2^ = 0.301).

The bootstrap analysis (Table 12) showed that the total effect of conscientiousness on anxiety was significant and negative (*β* = −0.347, *p* < 0.001). The total indirect effect was significant (*β* = −0.305, 95% CI [−0.344, −0.267]), whereas the direct effect was not, indicating full mediation by Red music cultural identification and self-identity. Among the three pathways, “conscientiousness → self-identity → anxiety” showed the strongest indirect effect (*β* = −0.253, 95% CI [−0.287, −0.220]). The serial pathway “conscientiousness → Red music cultural identification → self-identity → anxiety” was likewise significant (*β* = −0.112, 95% CI [−0.132, −0.092]), whereas the single-mediator pathway “conscientiousness → Red music cultural identification → anxiety” showed a positive indirect effect (*β* = 0.059, 95% CI [0.033, 0.084]).

**Table 12:** Bootstrap test of the mediation effects in the conscientiousness model.

Pathway		*β*	SE	95% CI lower	95% CI upper	Significance
Path 1	X2 → M1 → Y (Ind1)	0.059	0.013	0.033	0.084	*
Path 2	X2 → M2 → Y (Ind2)	−0.253	0.017	−0.287	−0.220	*
Path 3	X2 → M1 → M2 → Y (Ind3)	−0.112	0.009	−0.132	−0.092	*
	Total Indirect Effect	−0.305	0.019	−0.344	−0.267	*
	Direct Effect	−0.042	—	—	—	n.s.
	Total Effect	−0.347	—	—	—	*

X2, Conscientiousness; M1, Red Music Cultural Identification; M2, Self-Identity; Y, Anxiety; *β*, standardized effect estimate; n.s., not significant. Confidence intervals were obtained from 5,000 bias-corrected bootstrap resamples. * indicates a significant effect (95% CI does not include zero).

## 4 Discussion

Drawing on the culture–personality interaction framework, this study examined the relationships among personality traits, Red music cultural identification, self-identity, and adolescent anxiety in a sample of Chinese primary and secondary school students and tested a serial mediation model of “personality traits → Red music cultural identification → self-identity → adolescent anxiety.” The findings showed that neuroticism and conscientiousness shaped adolescents’ Red music cultural identification in opposite directions, with neuroticism negatively predicting and conscientiousness positively predicting cultural identification. Red music cultural identification, in turn, promoted self-identity development, whereas self-identity functioned as a core psychological resource buffering against anxiety. The serial mediation pathway was supported in both the neuroticism and conscientiousness models, although the underlying transmission patterns differed. These findings extend prior research by positioning Red music not only as a vehicle for ideological–political and esthetic education but also as an indigenous cultural–psychological variable relevant to adolescent mental health. The results further suggest that cultural resources may be converted into psychological resources through identity-related processes. The following sections discuss the mechanisms and implications of each pathway.

### 4.1 Predictive effects of personality traits on red music cultural identification

The study hypothesized that different personality dimensions exert differential predictive effects on adolescents’ Red music cultural identification. The results supported this hypothesis and further clarified the nature of these relationships: neuroticism significantly negatively predicted Red music cultural identification, whereas conscientiousness significantly positively predicted it.

The negative predictive effect of neuroticism on Red music cultural identification is consistent with the individual–culture interaction mechanism proposed by culture–personality interaction theory. Kardiner (1939) and Kardiner and Linton (1945) argued that personality traits shape how individuals engage with cultural institutions and internalize cultural values. From an integrative socioecological and gene–dynamic perspective, Lu et al. (2023) further characterized personality–culture relations as a bidirectional, dynamic process of mutual shaping. Similarly, Kitayama and Salvador (2024) emphasized that stable personality dispositions systematically moderate the depth of cultural-value internalization and that cultural participation constitutes an important pathway through which personality affects psychological outcomes. Individuals with high neuroticism are characterized by emotional instability, negative affectivity, and heightened threat sensitivity (Kotov et al., 2010; Racine et al., 2021), all of which may hinder the internalization of Red music cultural values through several mechanisms. First, difficulties in emotional regulation may reduce sustained affective engagement during esthetic participation, although emotional resonance is central to cultural identification formation (DeNora, 2000; Zhu, 2023). Second, heightened sensitivity to external stimuli and negative cognitive bias may interfere with constructive integration of the collectivist and patriotic values conveyed through Red music (McCrae and Costa, 1999; Soto and John, 2017). Schäfer and Mehlhorn's (2017) meta-analysis further demonstrated that individuals with different personality traits exhibit systematically different patterns of affective response and cognitive integration when exposed to the same musical-cultural stimuli, a pattern consistent with recent evidence on music-based interventions (de Witte et al., 2022), with highly neurotic individuals typically showing lower cultural engagement and weaker value internalization. These findings provide additional support for the present results.

The positive predictive effect of conscientiousness on Red music cultural identification is likewise theoretically grounded. Conscientiousness reflects self-regulation, responsibility, and achievement motivation (Costa and McCrae, 1992; Wilmot and Ones, 2019), a conceptualization that continues to be reaffirmed in current personality science (Bleidorn et al., 2022). Individuals high in conscientiousness tend to demonstrate stronger goal orientation, more constructive cognitive-processing strategies, and greater engagement in cultural participation. As a cultural symbol system with a clear value orientation, Red music conveys collectivism, patriotic sentiment, and a sense of historical mission that align closely with the responsibility, self-discipline, and achievement motivation characteristic of highly conscientious individuals (Kardiner and Linton, 1945; Lu et al., 2023). Consequently, students high in conscientiousness may be more likely to experience emotional resonance when engaging with Red music and to internalize its cultural values within their cognitive frameworks, thereby developing stronger cultural identification with Red music. This finding is consistent with Anglim et al.'s (2020) meta-analytic evidence showing that conscientious individuals exhibit stronger integration of adaptive psychological and behavioral tendencies associated with mental health, a conclusion reaffirmed in recent syntheses of personality psychology (Roberts and Yoon, 2022) and aligns with Schäfer and Mehlhorn's (2017) findings regarding systematic associations between personality traits and music-engagement behaviors, a pattern echoed in recent meta-analytic evidence on individual differences in music-based engagement (de Witte et al., 2022).

Notably, conscientiousness and neuroticism appear to influence Red music cultural identification through asymmetric mechanisms rather than simply operating in opposite directions. The self-discipline, responsibility, and achievement motivation characteristic of highly conscientious individuals show clear trait–value alignment with the collectivism, patriotism, and sense of historical mission conveyed through Red music, allowing cultural identification to emerge through direct value resonance. Neuroticism, by contrast, does not directly conflict with the core values of Red music, but may indirectly disrupt emotional resonance and cognitive integration through emotional instability, negative cognitive bias, and reduced capacity for esthetic engagement. Moreover, as Red music cultural identification is a culturally specific form of identification with strong indigenous characteristics (He et al., 2025), it may depend more heavily on active motivation for cultural participation and value internalization than merely on the absence of emotional instability. This possibility may partly explain the comparatively weaker predictive effect of neuroticism.

### 4.2 Predictive effects of red music cultural identification on self-identity

This study hypothesized that Red music cultural identification would positively predict self-identity. The results supported this hypothesis: Red music cultural identification significantly positively predicted self-identity in both the neuroticism and conscientiousness models, with consistent findings across the two models.

This finding aligns with the theoretical expectation that cultural identification contributes to self-identity development. According to social identity theory (Tajfel and Turner, 1986; Ward and Szabó, 2023), individuals derive a sense of self from the social groups to which they belong; group identification provides self-esteem, belonging, and security and is associated with higher subjective well-being. As a culturally distinctive form of identification with strong collective and historical characteristics, Red music cultural identification provides adolescents with cultural belonging and a stable value framework, enabling them to form clearer answers to core identity questions such as “Who am I?,” “Which group do I belong to?,” and “How am I connected to the social and historical world?” This process promotes self-identity integration and consolidation (Erikson, 1968; Branje, 2022). In their systematic review of adolescent identity development research over the past decade, Branje et al. (2021) noted that cultural context provides essential meaning-making resources for identity exploration and commitment, and that cultural belonging constitutes an indispensable dimension of contemporary adolescent identity development—a conclusion further supported by recent longitudinal work on identity dimensions (Umaña-Taylor and Rivas-Drake, 2021; Crocetti et al., 2023). These findings provide direct theoretical and empirical support for the cultural identification → self-identity pathway examined in this study.

Within the collectivist cultural context of China, the role of cultural identification in shaping self-identity may be particularly pronounced. Chen and Xu (2026) characterized cultural identification as the cognitive manifestation of cultural subjectivity—a process through which individuals define themselves and actively construct meaning within a cultural context. Therefore, Red music cultural identification is not merely an esthetic preference for a musical form but a process through which individuals internalize the historical memory, national spirit, and value orientations embedded in Red culture as part of the self. Strong identification with the values conveyed through Red music may provide adolescents with a stable value framework and sense of direction, reinforcing their understanding of social roles and historical responsibility and thereby promoting self-identity integration and consolidation (Li, 2024; Chen and Xu, 2026).

Correlational analyses further supported this finding: Red music cultural identification and self-identity showed one of the strongest positive correlations among all study variables. This indicates that their association was not only supported within the mediation model but also evident at the bivariate level. This pattern aligns with the music-identity literature reviewed in the Introduction. MacDonald et al.'s (2017) finding that musical identity facilitates adolescents’ self-concept integration received empirical support within the specific context of this study, consistent with the broader current literature on music, identity, and wellbeing (de Witte et al., 2022). Saarikallio's (2019) conceptualization of musical participation as a resource for emotional and identity regulation, together with Hargreaves et al.'s (2002) characterization of musical cultural identification as a dynamic dimension of the self— both of which continue to inform contemporary evidence on music, identity, and well-being (He et al., 2025) — are likewise corroborated within the indigenous cultural context of Red music.

The predictive effect of Red music cultural identification on self-identity was stronger in the neuroticism model than in the conscientiousness model. This difference may be attributable to the different control variables used in the two models. Specifically, neuroticism exerted a relatively strong negative effect on self-identity, yet Red music cultural identification retained a robust positive predictive effect, suggesting that its contribution to self-identity is at least partly independent of baseline personality characteristics. This result further supports the theoretical position that Red music cultural identification constitutes an independent psychological construct (He et al., 2025).

### 4.3 Predictive effects of self-identity on adolescent anxiety

The study hypothesized that self-identity negatively predicts anxiety in adolescents. The results supported this hypothesis: self-identity significantly negatively predicted adolescent anxiety in both the neuroticism and conscientiousness models, with directionally consistent findings across models.

This finding is consistent with the central propositions of identity theory and self-determination theory. Erikson (1968) argued that a stable sense of identity constitutes a core psychological resource for navigating the developmental challenges of adolescence, a proposition that continues to anchor current developmental research on adolescent identity and well-being (Branje, 2022). Individuals with coherent self-narratives and clearly defined value orientations tend to appraise external stressors more rationally, possess stronger emotional-regulation resources, and engage with life challenges more constructively, resulting in lower anxiety levels. Similarly, Deci and Ryan's (2000) self-determination theory proposes that fulfillment of the basic needs for autonomy, competence, and relatedness promotes mental health and reduces anxiety—an integrative view echoed in contemporary youth mental-health frameworks (McGorry et al., 2022). A stable student identity involves a clear understanding of one’s role as a student, a positive sense of school belonging, and active engagement with academic tasks, all of which may buffer the effects of academic and social-adjustment pressures on anxiety.

Conversely, identity diffusion reflects the absence of a stable self-referential framework. When exposed to uncertainty and external pressure, such individuals are more prone to loss of control and meaninglessness, increasing threat sensitivity and intensifying anxiety (Zeng et al., 2013; Crocetti et al., 2023). Marcia's (1966) identity status theory first proposed that adolescents in identity diffusion exhibit poorer mental health outcomes and significantly higher anxiety than peers who have achieved identity— a proposition whose empirical foundations Crocetti et al. (2023) have recently extended through longitudinal evidence on identity dimensions in adolescence and young adulthood. More recent research has refined this proposition from a dynamic-process perspective. Crocetti's (2017) three-dimensional model of commitment, exploration, and reconsideration of commitment, whose empirical foundations continue to expand in current identity science (Crocetti et al., 2023), together with Meeus's (2018) longitudinal re-examination of the identity-status continuum, whose conclusions have been consolidated in the most recent systematic review of identity development research (Maehler and Hernández-Torrano, 2025), confirmed that insufficient commitment during identity exploration is robustly and longitudinally associated with internalizing problems such as anxiety and depression. Branje et al.'s (2021) systematic review likewise emphasized that identity commitment is among the most consistent proximal psychological predictors of internalizing symptoms during adolescence, a conclusion reinforced by subsequent systematic evidence (Maehler and Hernández-Torrano, 2025). These conclusions are highly consistent with the present findings: higher self-identity scores were associated with lower anxiety levels.

Empirical research further supports these theoretical propositions. Wang and Yao (2021) found that students with stronger self-identity demonstrated greater psychological resilience and lower anxiety when facing academic challenges, consistent with recent longitudinal evidence on self-esteem, identity, and adolescent internalizing symptoms (Cai et al., 2025). A cross-cultural study by Vignoles et al. (2006) likewise showed that self-identity clarity and coherence are important predictors of mental health, a finding that continues to be corroborated in recent cross-cultural work on identity and well-being (Ward and Szabó, 2023). Maehler and Hernández-Torrano’s (2025) systematic review further confirmed that self-identity stability and coherence have important implications for mental health and social adaptation, with identity diffusion showing stable positive associations with internalizing problems such as anxiety and depression.

Notably, although the negative predictive effect of self-identity on anxiety was directionally consistent across the two models, its magnitude differed. The effect was substantially stronger in the conscientiousness model than in the neuroticism model. Self-identity functions as a core proximal mechanism linking personality traits and anxiety, although its observable effect is modulated by the shared variance between personality traits and anxiety. The protective effect of self-identity is more pronounced among adolescents with greater emotional stability.

### 4.4 The serial mediating role of red music cultural identification and self-identity

The study hypothesized that Red music cultural identification and adolescent self-identity would jointly serve as significant serial mediators between personality traits and anxiety; specifically, that the pathway “personality traits → Red music cultural identification → self-identity → adolescent anxiety” would be significant. The results supported this hypothesis: the serial mediation pathway (Path 3) was significant in both the neuroticism and conscientiousness models.

**Figure 1:**
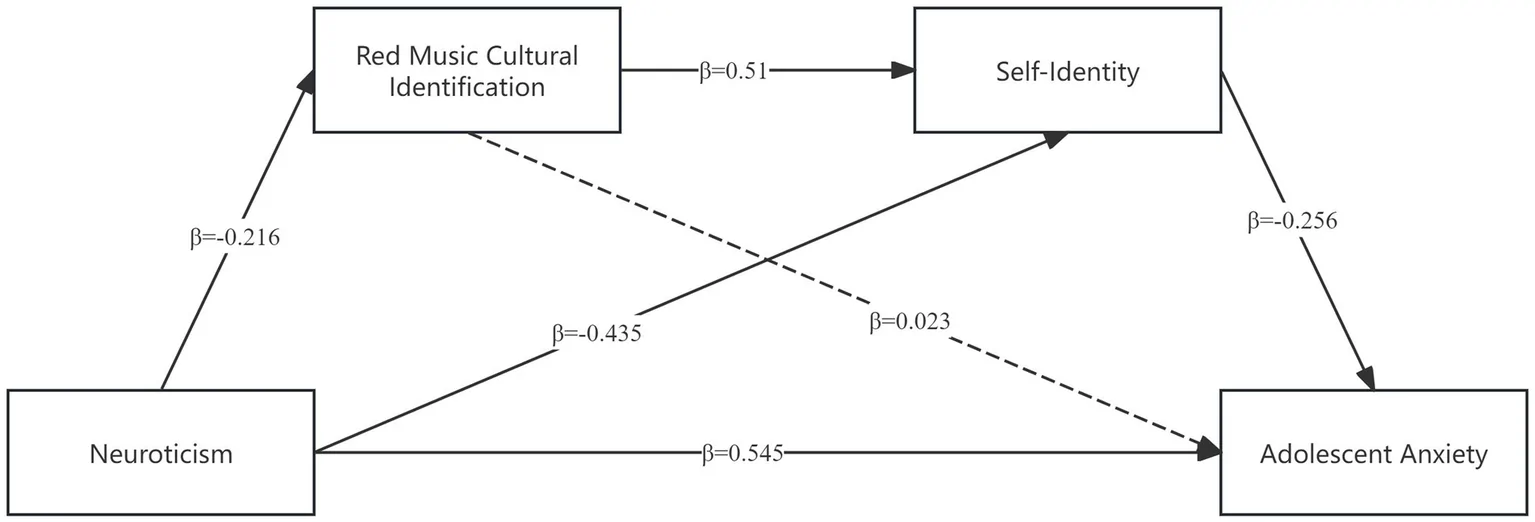
Serial mediation model of the relationship between neuroticism and adolescent anxiety through Red music cultural identification and self-identity.

Red music cultural identification and self-identity functioned as significant sequential mediators between neuroticism and anxiety. Specifically, the indirect effect of Path 1 (neuroticism → Red music cultural identification → anxiety) was not significant, indicating that Red music cultural identification alone was insufficient to mediate the relationship between neuroticism and anxiety. By contrast, the indirect effect of Path 2 (neuroticism → self-identity → anxiety) was significant and showed the largest effect size among the three indirect pathways. The serial mediation effect of Path 3 (neuroticism → Red music cultural identification → self-identity → anxiety) was likewise significant. Based on these findings, the study constructed a serial mediation model in which neuroticism influences anxiety through Red music cultural identification and self-identity (see Figure 1).

This pattern of findings suggests an important psychological mechanism through which neuroticism may influence adolescent anxiety. The positive indirect effect observed in the neuroticism model does not indicate that Red music cultural identification and self-identity increase anxiety. Rather, neuroticism appears to indirectly amplify anxiety by weakening these two psychological resources. The emotional instability characteristic of highly neurotic individuals may substantially weaken identification with Red music culture. Reduced cultural identification, however, does not appear to influence anxiety directly; instead, its effects operate through self-identity, a core self-related construct, before ultimately affecting anxiety. This finding suggests that Red music cultural identification functions as a distal cultural resource whose mental health effects are expressed primarily through identity integration processes (DeNora, 2000; Hargreaves et al., 2002; de Witte et al., 2022). From the perspective of culture–personality interaction theory (Kardiner and Linton, 1945; Kitayama and Salvador, 2024), highly neurotic individuals may struggle to internalize cultural values because of emotional-regulation difficulties, resulting in lower Red music cultural identification, reduced cultural support for identity development, and ultimately elevated anxiety. This serial pathway further supports the proposition that cultural identification does not influence mental health independently but exerts its protective psychological effects through the shaping of self-identity (Erikson, 1968; Tajfel and Turner, 1986; Ward and Szabó, 2023).

**Figure 2:**
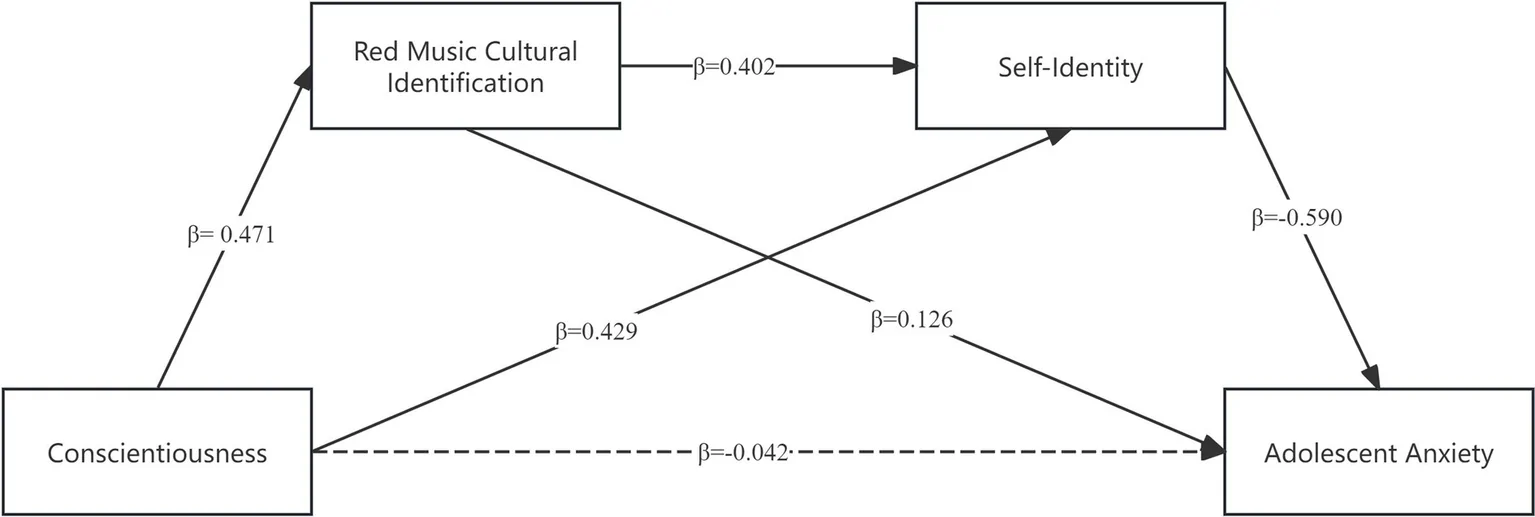
Serial mediation model of the relationship between conscientiousness and adolescent anxiety through Red music cultural identification and self-identity.

The conscientiousness model exhibited a pattern distinct from that of the neuroticism model (see Figure 2). The indirect effect of Path 1 (conscientiousness → Red music cultural identification → anxiety) was significant but small and positive in direction. The indirect effect of Path 2 (conscientiousness → self-identity → anxiety) was significant and represented the strongest protective pathway. The serial mediation effect of Path 3 (conscientiousness → Red music cultural identification → self-identity → anxiety) was likewise significant and negative.

First, the influence of conscientiousness on anxiety was fully mediated by the mediating variables, indicating that the anxiety-buffering effect of conscientiousness operates indirectly through the promotion of cultural identification and self-identity. This finding is consistent with evidence from Roberts et al. (2014) and Wilmot and Ones (2019), who showed that conscientiousness affects long-term mental health through psychosocial mechanisms—a proposition reinforced by recent global evidence on youth mental health (McGorry et al., 2022). Second, the positive indirect effect observed in Path 1 may initially appear counterintuitive and therefore warrants closer examination. Statistically, this pattern reflects a suppression effect. Psychologically, it may indicate that strong cultural identification can also involve heightened responsibility or self-monitoring pressure. When individuals strongly identify with the values associated with Red music culture but have not yet integrated them into a stable self-concept, the accompanying sense of responsibility and perceived social expectations may temporarily increase self-imposed standards and normative pressure, thereby slightly elevating anxiety (Chen and Xu, 2026; Luo and Chen, 2026). However, when this cultural identification becomes integrated into a stable self-identity, self-identity exerts a substantial anxiety-buffering effect, causing the serial mediation pathway overall to function protectively.

Neurophysiological evidence from He et al. (2026) offers complementary support for these findings. Using the SC-IAT paradigm with event-related potentials, they showed that Red song exposure elicited multi-stage neural differences from neutral music (N1, P2, N3, and LPC components), and that its effects on implicit prosocial attitudes were modulated by individual-level factors rather than operating through a uniform direct pathway. Two parallels with the present findings are noteworthy. First, both studies suggest that Red music engagement influences downstream psychological outcomes only through integration with individual-level processes—music training experience in He et al. (2026) and self-identity formation in the present study. Second, the ERP evidence that Red music engagement recruits automatic, pre-conscious processing pathways provides a plausible neurocognitive foundation for the indirect pathway from Red music cultural identification to anxiety through self-identity, suggesting that its psychological benefits unfold through automatic activation of self-relevant cognitive schemas rather than through explicit reflective processes alone.

Collectively, these findings highlight the complexity of the relationship between cultural identification and mental health and distinguish the study from simplistic accounts that portray cultural identification as uniformly beneficial. The psychological effects of cultural identification are not linear or automatic; rather, they appear to depend partly on whether cultural identification becomes integrated into the self (Vignoles et al., 2006; Chen and Xu, 2026). In the context of Red music cultural education, an important consideration may be not only increasing students’ familiarity with or endorsement of Red music but also helping them develop more stable identity-based belonging and personal meaning through esthetic experience, emotional resonance, and value understanding (MacDonald et al., 2017; Han and Guo, 2023).

### 4.5 Limitations and future directions

First, the study employed a cross-sectional survey design, which is known to yield potentially biased estimates of longitudinal mediating processes (Maxwell and Cole, 2007), a concern reaffirmed in recent methodological work showing that cross-sectional mediation models can systematically misrepresent underlying temporal dynamics (VanderWeele, 2023). Future research would benefit from longitudinal or cross-lagged panel designs to clarify the temporal dynamics linking personality traits, Red music cultural identification, self-identity, and adolescent anxiety.

Second, although the sample was relatively large, recruitment may have been shaped by regional differences, school type, and educational resources, limiting generalizability—a well-recognized concern in psychological research since Henrich et al. (2010) and one that continues to be documented in recent cross-cultural adolescent research showing systematic sample-based biases (Nielsen et al., 2024). Future research should broaden the geographic scope of sampling and examine model stability across sex, school stage, urban–rural background, and prior musical experience.

Third, although the two self-developed instruments demonstrated satisfactory structural, convergent, and discriminant validity, their criterion-related validity was not examined against external reference measures. Construct validation has long required convergence with theoretically related criteria (Cronbach and Meehl, 1955), and recent methodological reviews continue to identify insufficient criterion-related evidence as a pervasive weakness in newly developed scales (Lambert and Newman, 2023). Future research should incorporate established criterion measures—such as the National Identity Scale, the Patriotism Scale, and general cultural identification instruments—and employ stratified sampling to enable known-groups validation.

Finally, several theoretically relevant psychological variables—such as sense of responsibility, emotion-regulation strategies, self-esteem, and empathy—were not included. Omitting relevant mediators is a long-standing source of bias in mediation inference (MacKinnon et al., 2007), and recent longitudinal research on adolescent internalizing symptoms continues to demonstrate that emotion regulation and self-esteem operate as robust proximal predictors that mediation models of anxiety should incorporate (Cai et al., 2025). Future research should construct more comprehensive moderated-mediation models to examine how multiple psychological resources jointly shape adolescent anxiety.

## 5 Conclusion

This study aimed to test, within the culture–personality interaction framework, whether the association between Big Five personality traits and adolescent anxiety is sequentially mediated by Red music cultural identification and self-identity, and the results largely corroborated the four hypotheses: neuroticism negatively and conscientiousness positively predicted Red music cultural identification, Red music cultural identification positively predicted self-identity, self-identity negatively predicted anxiety, and the serial pathway “personality traits → Red music cultural identification → self-identity → adolescent anxiety” was significant in both the neuroticism and conscientiousness models.

By incorporating Red music cultural identification as an independent psychological variable into a predictive model of adolescent anxiety, this study extends prior work that has largely framed Red music in ideological–political or esthetic-education terms. By integrating cultural identification and self-identity within a single serial-mediation framework, it further clarifies a pathway through which cultural resources may be converted into mental-health advantages: cultural identification appears to exert its protective effects primarily through identity-integration processes rather than through a direct route.

Adolescent mental health interventions may benefit from integrating personality traits, cultural identification, and self-identity within a broader systemic framework rather than targeting each variable in isolation. For students high in neuroticism, interventions may focus on strengthening emotional-regulation capacities and supporting identity stability. For students high in conscientiousness, greater attention may be needed to help transform cultural-value identification into constructive resources for identity integration while avoiding excessive responsibility and self-monitoring pressure. If Red music cultural education is meaningfully integrated with adolescents’ identity-development processes, it may serve as a useful esthetic-education approach for promoting adolescent mental health.

## Data Availability

The original contributions presented in the study are included in the article/supplementary material, further inquiries can be directed to the corresponding author.
